# The complete chloroplast genome sequence of *Ferula sinkiangensis* K. M. Shen, a precious and endangered traditional Chinese medicine

**DOI:** 10.1080/23802359.2021.1927869

**Published:** 2021-05-19

**Authors:** Congzhao Fan, Guoping Wang, Yuanjin Qiu, Yaqin Zhao, Jizhao Zhang, Jingyuan Song, Xiaojin Li

**Affiliations:** a Xinjiang Institute of Chinese Materia Medica and Ethnical Materia, Xinjiang Key Laboratory of Chinese Materia Medica and Ethnic Materia Medica, Urumqi, People’s Republic of China; b Key Lab of Chinese Medicine Resources Conservation, State Administration of Traditional Chinese Medicine of the People's Republic of China, Institute of Medicinal Plant Development, Chinese Academy of Medical Sciences and Peking Union Medical College, Beijing, People’s Republic of China

**Keywords:** Chloroplast genome, *Ferula sinkiangensis*, Illumina sequencing, phylogenetic analysis

## Abstract

*Ferula sinkiangensis* K. M. Shen is a valuable traditional Chinese medicine historically used to treat stomachache and rheumatoid arthritis. The chloroplast genome of *Ferula* genus plant has not been previously reported. This study reported the complete chloroplast genome sequence of *F. sinkiangensis* based on high-throughput sequencing. The genome was 166,583 bp in length, containing a small single-copy (SSC) region of 17,595 bp and a large single-copy (LSC) region of 85,242 bp, separated by two inverted repeats (IRs) of 31,873 bp, each. The genome contained 114 unique genes, including 80 protein-coding genes (PCGs), four rRNA genes, and 30 tRNA genes. In addition, 17 genes contained one or two introns, including nine PCG genes with a single intron, two PCG genes harboring two introns, and six tRNA genes harboring a single intron. In this study, *F. sinkiangensis* K. M. had the closest genetic relationship with *Torilis scabra* and clustered with the *Umbelliferae* family species.

The *Ferula* resin is used globally as a traditional medicinal herb, and Chinese Pharmacopoeia recently documented *Ferula sinkiangensis* as one of the main primitive plant. The resin of *F. sinkiangensis* is used to treat many diseases, especially stomachache, flatulence, and poor digestion (Iranshahy and Iranshahi 2011; Mahendra and Bisht 2012). Modern pharmacology and biological studies have shown that *F. sinkiangensis* has antiviral (Nazari and Iranshahi 2011), antifungal (Kavoosi et al. 2013), cancer chemopreventive, and anti-influenza effects (Iranshahi et al. 2010; Zhou et al. 2017).

In China, the *Ferula* genus is mostly distributed in Xinjiang but has been destroyed due to excessive collection. In addition, slow growth, change in reclamation, irrigation, road construction, and deterioration of the native habitat contribute to the annual shrinkage of *Ferula* resources. Currently, wild resources of *F. sinkiangensis* are extremely endangered; thus, many researchers are interested in studying its endangerment mechanism to protect and utilize it. This study assembled the complete chloroplast genome of this species by Illumina sequencing (San Diego, CA) to facilitate its phylogenetic analysis, conservation genetics, and ensure sustainable utilization. The genome sequence data that support the findings of this study are openly available in GenBank of NCBI at https://www.ncbi.nlm.nih.gov/ under the accession MW411057. The associated BioProject, SRA, and Bio-Sample numbers are PRJNA715948, SUB9326528, and SAMN18388803, respectively.

The number of species in the *Umbelliferae* family with published chloroplast genomes is relatively small. In this study, the chloroplast genome of a *Ferula* genus plant was assembled for the first time. The determination of this complete plastid genome sequence is a useful resource and provides molecular data to illuminate the *F. sinkiangensis* evolution and its endangerment mechanism.

In this study, fresh leaves of *F. sinkiangensis* were collected from Baishidun Township of Yining City (82.0574 E, 43.6743 N), Xinjiang, China. The voucher specimen (654021120525001) was deposited at the Xinjiang Institute of Chinese Materia Medica and Ethnical Materia. The genomic DNA was extracted using the DNeasy Plant Mini Kit (QIAGEN, Valencia, CA). High-throughput sequencing was conducted using the Illumina HiSeq X Ten platform, and data processing was according to Yan et al. (2019).

The complete chloroplast genome was annotated using *Torilis scabra* (MN105615) as a reference. The chloroplast genome of *F. sinkiangensis* has a circular quadripartite structure which is similar to major angiosperm chloroplast genomes (Hahn C et al. 2013). It has a length of 166,583 bp, contained a small single-copy (SSC) region of 17,595 bp and a large single-copy (LSC) region of 85,242 bp separated by two inverted repeats (IRs) of 31,873 bp, each. Similar to the chloroplast genomes of other higher plants, the base composition is asymmetric (30.7% A, 19% C, 18.9% G, and 31.4% T) with an overall GC content of 62.1%.

The *F. sinkiangensis* chloroplast genome contained 114 unique genes, including 80 protein-coding genes (PCGs), four rRNA genes, and 30 tRNA genes. In addition, 17 genes contain one or two introns, which include nine PCG genes (atpF, ndhA, ndhB, petB, rpl2, rpl16, rpoC1, rps12, rps16) possessing a single intron, two PCG genes (clpP, ycf3) harboring two introns, and six tRNA genes (trnA-UGC, trnG-UCC, trnI-GAU, trnK-UUU, trnL-UAA, trnV-UAC) harboring a single intron.

A neighbor-joining phylogeny was constructed using MEGA7.0 (Kumar et al. 2016) based on nine genes (accD, atpA, atpB, matK, psbB, psbD, rbcL, rpoA, rpoC1) in the chloroplast genome of *F. sinkiangensis* and 42 other species from the *Umbelliferae* family. The phylogenetic tree analysis showed that *F. sinkiangensis* was closely related to the *T. scabra* (Figure 1).

**Figure 1.**
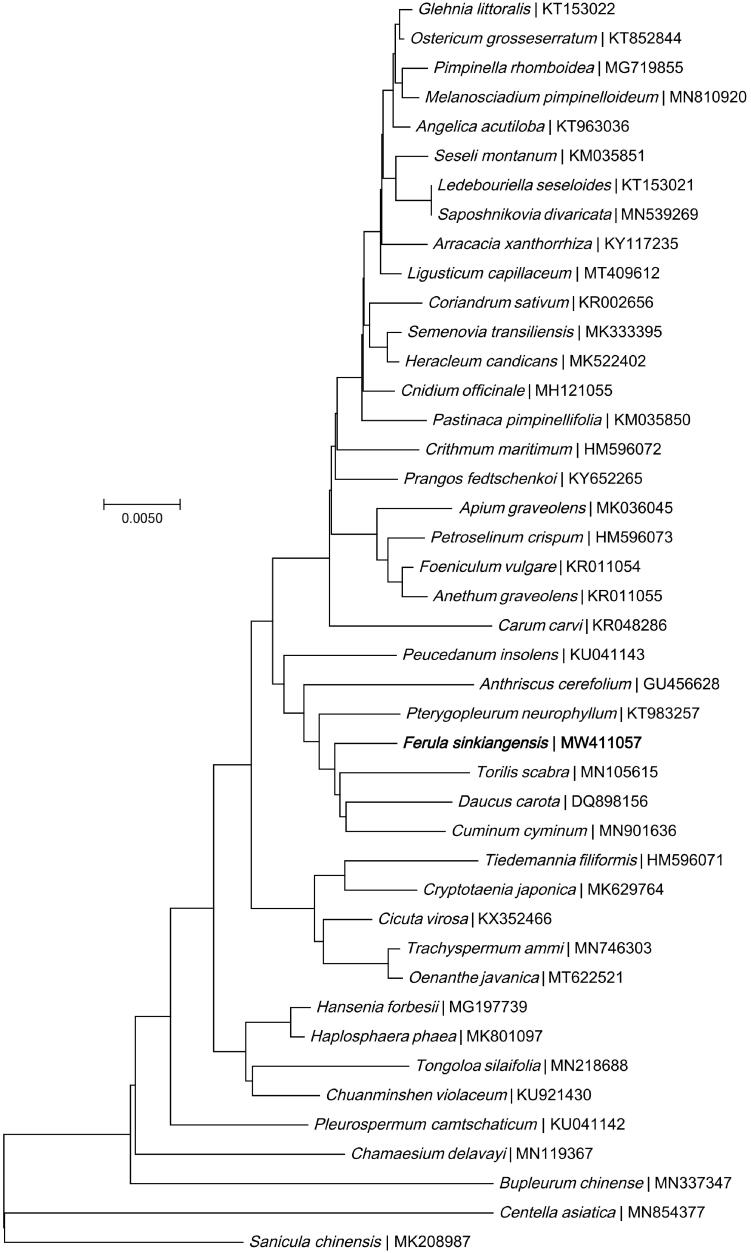
Phylogenetic relationships of 43 *Umbelliferae* family species based on the neighbor-joining analysis of chloroplast genes. The bootstrap values were based on 1000 replicates, and are shown next to the branches.

## Data Availability

We confirm that the data supporting the findings of this study are available within the article and its supplementary materials. The data that support the study findings have been deposited in GenBank (https://www.ncbi.nlm.nih.gov/) under accession number MW411057.
